# Relationships between surrogate measures of mechanical and psychophysiological load, patellar tendon adaptations, and neuromuscular performance in NCAA division I men's volleyball athletes

**DOI:** 10.3389/fspor.2023.1065470

**Published:** 2023-02-22

**Authors:** Brian M. Guthrie, Erica L. King, Shriniwas Patwardhan, Qi Wei, Siddhartha Sikdar, Parag V. Chitnis, Margaret T. Jones

**Affiliations:** ^1^ Patriot Performance Laboratory, Frank Pettrone Center for Sports Performance, George Mason University, Fairfax, VA, United States; ^2^ Department of Bioengineering, George Mason University, Fairfax, VA, United States; ^3^ Center for Adaptive Systems of Brain-Body Interactions, George Mason University, Fairfax, VA, United States; ^4^ Sport, Recreation, and Tourism Management, George Mason University, Fairfax, VA, United States

**Keywords:** tendinopathy, jumps, workload, ultrasound, injury

## Abstract

**Introduction:**

Patellar tendon adaptations occur in response to mechanical load. Appropriate loading is necessary to elicit positive adaptations with increased risk of injury and decreased performance likely if loading exceeds the capacity of the tendon. The aim of the current study was to examine intra-individual associations between workloads and patellar tendon properties and neuromuscular performance in collegiate volleyball athletes.

**Methods:**

National Collegiate Athletics Association Division I men's volleyball athletes (*n* = 16, age: 20.33 ± 1.15 years, height: 193.50 ± 6.50 cm, body mass: 84.32 ± 7.99 kg, bodyfat%: 13.18 ± 4.72%) competing across 9 weeks of in-season competition participated. Daily measurements of external workloads (i.e., jump count) and internal workloads [i.e., session rating of perceived exertion (sRPE)] were recorded. Weekly measurements included neuromuscular performance assessments (i.e., countermovement jump, drop jump), and ultrasound images of the patellar tendon to evaluate structural adaptations. Repeated measures correlations (*r-rm)* assessed intra-individual associations among performance and patellar tendon metrics.

**Results:**

Workload measures exhibited significant *negative small* to *moderate* (*r-rm *=−0.26–0.31) associations with neuromuscular performance, *negative* (*r-rm *= −0.21–0.30), and *positive* (*r-rm *= 0.20–0.32) *small* to *moderate* associations with patellar tendon properties.

**Discussion:**

Monitoring change in tendon composition and performance adaptations alongside workloads may inform evidence-based frameworks toward managing and reducing the risk of the development of patellar tendinopathy in collegiate men's volleyball athletes.

## 1. Introduction

Athlete monitoring frameworks have been developed to manage fatigue, optimize training prescription, and mitigate injury risk specific to a sport. Volleyball athletes performing high volumes of forceful, cyclic movement place large stress and strain on the knee resulting in a high prevalence of patellar tendinopathy (1–4). Risk factors for developing patellar tendinopathy include: poor movement kinetics and kinematics (5), limited hip and ankle range of motion (6), body weight (7), quadriceps and hamstrings flexibility (7), vertical jump performance (1), and volume of training (1, 7). Greater hip and knee flexion may serve as compensatory patterns to minimize pain during the movement (8). Research involving volleyball athletes with patellar tendinopathy showed higher adduction and internal rotation moments during take-off and landing of spike jumps (9). Height of landing and fatiguing conditions may play a role in developing a movement strategy producing indicators of knee valgus (10). Therefore, load management is an important part of athlete monitoring in volleyball players.

The mechanical and psychophysiological load-response pathways describe the external and internal athlete loads and subsequent adaptations, respectively (5, 11). External load is characterized as the amount of workload an athlete does while internal load describes the physiological or psychophysiological response to a given external stimulus. Psychophysiological load is the culmination of the psychological and physiological stress response experienced by an athlete that is an immediate response or can be a surrogate measure if assessed post-training (11, 12). Accumulated fatigue due to excessive loads or inadequate recovery may result in performance decrements or higher risk of injury (13). In particular, overuse injury is commonly represented as a mechanical fatigue phenomenon, thus, monitoring performance and structural adaptations may be of interest (14). In collegiate women volleyball athletes, a significant association between increased sets played and decreased countermovement jump height was established across a competitive season (15). These findings were attributed to more time spent in the eccentric phase and greater peak rate of force development providing evidence that altered jump strategy may be indicative of fatigue (16–19). Compared to the countermovement jump, drop jump kinetics and kinematics may differ following stretch shortening cycle fatigue due to the temporal constraints of the movement (20, 21). While ample evidence exists in support of altered kinetics and kinematics with increased loads (15, 17, 18), research investigating tissue-specific adaptations to load fluctuations is limited.

A pathological tendon presents structural abnormalities such as dimensions, organization and arrangement of collagen fibers, neovascularization, and stiffness, which can be assessed using imaging modalities like ultrasound (22–24). In elite junior volleyball players, symptomatic tendons were characterized as hypoechoic (i.e., reduced mean grayscale value indicating disorganized collagen bundle) with increased neovascularization (25), increased tendon width (25), greater cross-sectional area (26) and lower stiffness (26). Stiffness is the resistance to deformation with a given application of force commonly assessed using ultrasound elastography; however, this method requires specialized equipment and expertise. Hand-held myotonometry, which administers a low amplitude impulse to the tissue and analyzes the resulting accelerations, is a non-invasive, relatively inexpensive, and efficient method of assessing viscoelastic properties of muscle tissue (27) and mechanical properties of tendons. A stiffer tendon improves load tolerance resulting in resilience to tissue damage and enhanced force transmission (28, 29). Decrements in stiffness may signal compromised tendon structure possibly leading to impaired performance and higher risk of injury development. Therefore, monitoring changes in structural and mechanical properties of patellar tendons may inform efforts to detect the onset of tendon overuse.

There have been few reports of longitudinal monitoring of tissue characteristics. For example, in male volleyball athletes, increases in external and internal loads were associated with changes in ultrasound tissue characterization toward a less healthy tendon (30). Further, in professional handball athletes, similar changes in ultrasound tissue characterization of the patellar tendon were reported across phases of competition (31). The utility of monitoring structural changes has been in question due to disassociated time course of symptom resolution and improvements in tissue structure (32, 33)..

However, clinical impairments in lower extremity neuromuscular function may persist after symptom resolution (34). In addition, asymptomatic abnormalities are a risk factor for the development of future symptoms (35) and may alter neuromuscular function (30, 34) thereby warranting further investigation into the management of volleyball athlete load and monitoring of tendon structure. To date, no studies have examined the mechanical or psychophysiological load-response pathways in collegiate volleyball using neuromuscular performance and combined ultrasound and myotonometry as response variables. Further, the existing evidence examining the utility of ultrasound and myotonometry is inconclusive. Therefore, the overall purpose was to examine associations within the mechanical and psychophysiological load-response pathway between athlete loads, patellar tendon properties, and neuromuscular performance in collegiate men volleyball athletes. It was hypothesized that associations exist between external workload and tendon properties as well as between external and internal workload and neuromuscular performance.

## 2. Materials and methods

### 2.1. Study design

**Figure 1:**
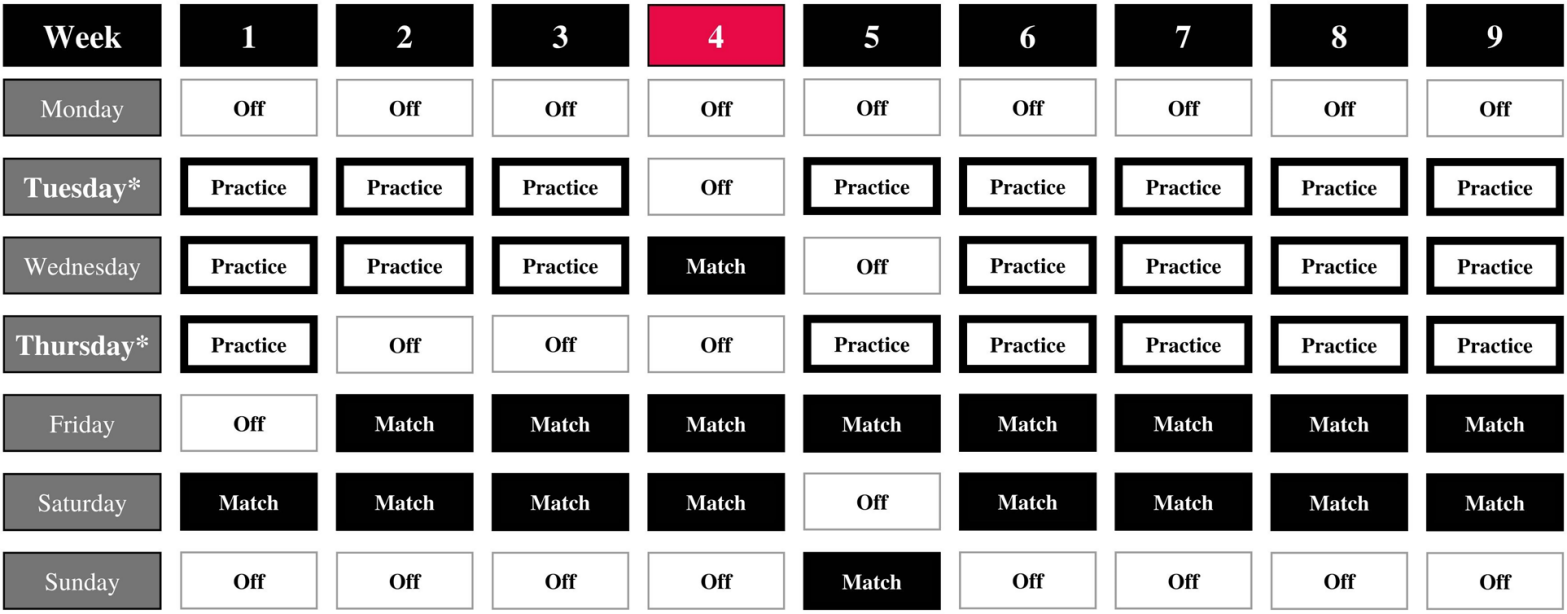
Data collection schedule. Red fill indicates cross-country travel with missing data for all participants.

A cohort of collegiate men's volleyball athletes were monitored across the competitive sport season to identify neuromuscular performance and tendon adaptations of the knee. External and internal loads were assessed for each practice and match. The training and competition schedule for included time points is represented in Figure 1. Participants arrived at the laboratory on the same day each week between 7:00–11:00 am and had refrained from physical exercise. In addition to sport practices, athletes participated in the same periodized resistance training program led by a Certified Strength and Conditioning Specialist^®^. Upon arrival to the laboratory, standardized locations on the patellar tendon were marked for each participant. Tissue properties were assessed using ultrasonography to quantify tendon thickness and echogenicity, and myotonometry to quantify tendon stiffness. Next, participants were instructed to perform a supervised ∼10 min standardized warm-up, which consisted of dynamic exercises with incrementally increasing intensity and ending in maximal effort practice trials of the tested movements. Data collection began with the countermovement jump and concluded with the drop vertical jump. Countermovement jump variables included jump height, peak force, and reactive strength index-modified (RSImod) while drop jump variables included jump height, peak force, and reactive strength index (RSI).

### 2.2. Participants

Sixteen NCAA Division I men's volleyball athletes (age: 20.3 ± 1.2 years, height: 193.5 ± 6.5 cm, bodyfat %: 13.18 ± 4.72%, body mass: 84.3 ± 8.0 kg) participated in the data collection. The inability to participate in maximal effort performance testing throughout the course of the data collection excluded one athlete from participation. Players were medically cleared for intercollegiate athletic participation by the University's sports medicine staff, had the risks and benefits explained to them beforehand, signed an institutionally approved consent form to participate, and completed a medical history form. This study was conducted according to the Declaration of Helsinki guidelines and procedures were approved by the University's Institutional Review Board for use of human subjects in research.

### 2.3. Measurements and instrumentation

#### 2.3.1. Athlete load monitoring

##### 2.3.1.1. External load

Participants were outfitted with wearable inertial measurement units (VERT; Mayfonk Inc., Ft. Lauderdale, FL, United States) embedded with a 3-axis accelerometer and 3-axis gyroscope in order to detect vertical displacements. Units and participants received a unique identifier to ensure the same device was worn each practice session and match across the season. Each session began with a team-led dynamic warm-up and ended following completion of the last drill in practice or once the final point was scored in match play. The raw signal was processed using proprietary algorithms then saved in cloud-based storage. Jump count was extracted as a surrogate measure of external workload. Active minutes were quantified using accelerometry from the device.

##### 2.3.1.2. Internal load

Following every training session and match, participants provided a rating of perceived exertion (i.e., Borg CR-10 scale) as to session or match difficulty. The Borg CR-10 scale is a validated and reliable scale for use with athletic populations as a surrogate measure of internal load (36, 37). Further, session rating of perceived exertion (sRPE) was calculated using the following equation:

(1) sRPE=RPE×duration

Duration of the session was individualized by reporting active minutes (38). The researcher approached each participant within 30 min of completion of the session and showed them the scale with corresponding verbal anchors (37). The participants were instructed to indicate the value by pointing to ensure confidentiality and prevent influence on other participants (39, 40).

##### 2.3.1.3. Workload efficiency

Determining the relationship between external and internal load has been established as a useful assessment for indicating a change in perceived demands to a given external stimulus (41). Workload efficiency describes the ratio between external and internal workload with a higher ratio indicating high tolerance to a given workload compared to a lower ratio. This was calculated by dividing jump count by the sRPE for each day of training and competition.

#### 2.3.2. Patellar tendon properties

In an effort to maintain consistency, all measurements of the patellar tendon using ultrasonography and myotonometry were conducted by the same researcher. The location of assessment was standardized at the beginning of each session by marking each participant at pre-determined locations of the tissue structure.

##### 2.3.2.1. Ultrasonography

**Figure 2:**
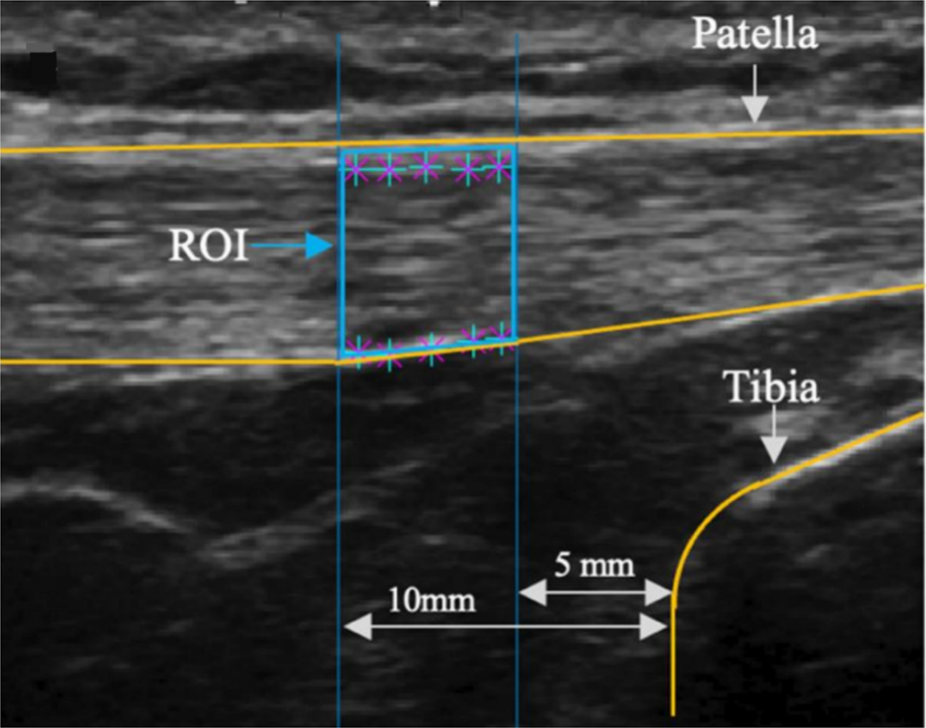
Ultrasound image of patellar tendon. ROI, region of interest.

Two-dimensional B-mode ultrasound technology (eSaote BioSound MyLab 25, Biosound Esaote, Inc., Indianapolis, IN, United States) was used with a 5 cm linear transducer (frequency, 7.5 MHz; axial resolution < 0.5 mm) to assess dimensions and quality of the patellar tendon. Ultrasound settings remained consistent across participants and timepoints. To acquire images of the patellar tendon in a lengthened position, participants assumed a seated position with the knee resting at 90°. Ultrasound images were post-processed using a custom MATLAB script (MathWorks, Natick, MA, United States). The region of interest of the patellar tendon (Figure 2) was determined by taking the average thickness at multiple points between 5 and 10 mm away from the tibial bone. Echogenicity was determined by the mean pixel value of the region of interest in the images collected.

##### 2.3.2.2. Myotonometry

A hand-held myotonometer (MyotonPro, Myoton Ltd., Estonia), which has been shown to be a valid and reliable instrument to measure viscoelastic properties of lower extremity tissue structure (27) assessed stiffness of the biological tissue. Stiffness was measured using the equation:

(2) $$\mathrm{Stiffness}(N/m)=a_{\max}\cdot m_{\mathrm{probe}}/\Delta l$$

where $a_{\max}$ is the acceleration of the damped oscillation, $m_{\mathrm{probe}}$ is the mass of the measurement mechanism and Δl is the probe displacement (42). To compare the myotonometric variables, measurements were taken in the position corresponding to the aforementioned ultrasound imaging protocol.

#### 2.3.3. Neuromuscular performance

Lower extremity neuromuscular performance was assessed by ground reaction forces from countermovement jumps and drop vertical jumps. Data were collected using a portable force platform (AccuPower; AMTI, Watertown, MA, United States) with a sampling rate of 1600 Hz and equipped with a data acquisition system utilizing custom LabVIEW (National Instruments, Austin, TX, United States). Trials were further analyzed with a custom MATLAB script using a 4th order, low pass, bidirectional Butterworth filter (43). Participants performed three maximal effort trials for both tests and were instructed to jump as high and as fast as possible while keeping their hands on their hips. They completed 3 trials with up to 2 additional trials if the trial was not performed correctly. Two familiarization sessions were completed prior to the actual data collection.

##### 2.3.3.1. Countermovement jump

Participants began the assessment from a tall standing position with hands on their hips. Researchers cued participants to perform a rapid countermovement to a self-selected depth of approximately 90° of knee flexion followed by an immediate rapid vertical jump.

**Figure 3:**
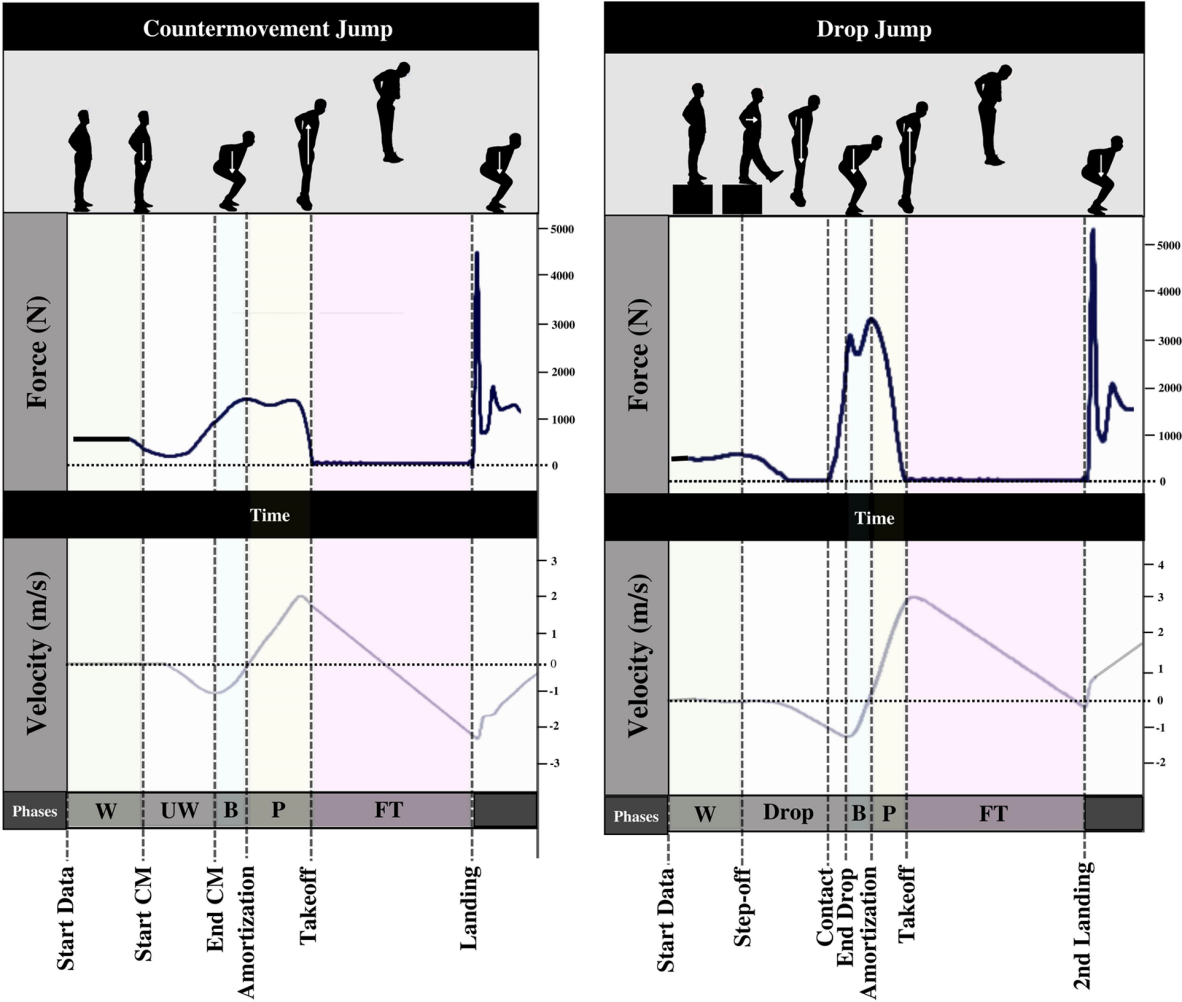
Phase detection of countermovement jump and drop vertical jump. CM, countermovement; W, weighting phase, UW, unweighting phase; B, braking phase; *P*, propulsive phase; FT, flight time.

Phases of the countermovement jump were identified by locating specific timepoints throughout the force-time and velocity-time curves (Figure 3). The weighing phase began at the start of the data acquisition to the initiation of the countermovement. The point in time that force fluctuated above or below body weight ±5 standard deviations was representative of the start of the countermovement. Takeoff was determined as the first timepoint that force dropped below 20 N. The unweighting phase was identified as the start of the countermovement to the timepoint of minimum velocity between the start of the movement and takeoff. The braking phase was identified as the end of the unweighting phase to the timepoint that velocity exceeded zero. The propulsive phase began at the end of the braking phase to the time of takeoff. The flight phase began at takeoff to the last timepoint force was below the 20 N threshold. Body weight was measured as the mean force of the first 500 data points of the weighing phase. Jump height was measured using the following equation:

(3) $$\mathrm{Jump}\;\mathrm{Height}(\mathrm{cm})={\left(\frac{9.81*\mathrm{Flight}\;\mathrm{Time}}{2}\right)}^{2}\;$$

The RSImod was calculated by dividing countermovement jump height by the time to takeoff, which was defined as the time from the start of the countermovement to the time takeoff occurred. Peak concentric force was calculated by the maximum force attained in the propulsive phase.

##### 2.3.3.2. Drop vertical jump

Participants began the assessment from a tall standing position atop a 30 cm plyometric box with hands on their hips. Researchers cued participants to step directly forward off the box (i.e., self-selected foot) with no downward translation of their center of mass then regain bilateral posture and land with both legs simultaneously upon ground contact. Further, participants were instructed to minimize the time of contact with the force platform and maximize time spent in the air.

Except for initiation of the drop jump, phases were identified as previously described for the countermovement jump. The drop off the box was used to determine the onset of the first landing by finding the timepoint that force increased beyond 20 N after the drop. Variables extracted from the drop vertical jump force-time curve included jump height, peak concentric force, andRSI. RSI is calculated by dividing the drop vertical jump height by the contact time. Contact time was defined as the time between initial contact and takeoff.

### 2.4. Statistical analysis

Data analysis was performed using statistical software (R Studio, version 1.4.1717, https://www.r-project.org). Descriptive statistics are reported as mean ± standard deviation. Intra-class correlation coefficients (ICC) and standard error of measurement (SEM) were used to assess intra-rater reliability for all patellar tendon measurements. To assess intra-rater reliability, the two-way mixed effects, absolute agreement, and multiple rater/measurements model was evaluated using the following formula (44):

(4) $$\mathrm{ICC}(3,k)=\frac{MS_{R}-MS_{E}}{MS_{R}+\frac{MS_{C}-MS_{E}}{n}}$$

The estimated ICC and the 95% confidence internal were reported. ICCs were interpreted as: Poor (<0.5), moderate (0.5–0.75), good (0.75–0.9), excellent (>0.9) (45). SEM was calculated using the following formula:

(5) $$\mathrm{SEM}={SD}_{pooled}\times \sqrt{1}-\mathrm{ICC}$$

Repeated measures correlations (RMCORR) were assessed using the *rmcorr* package. RMCORR is a robust analysis strategy enabling the assumption of independence to be relaxed in a repeated measures design (46). RMCORR is also robust to missing data representing conceptual similarity to a multilevel null model allowing for random intercepts while evaluating common slopes representing intra-individual associations (46). Considering these advantages, RMCORR better represents the research question compared to repeated measures analysis of variance (ANOVA) or simple linear regression or correlation, which defers to aggregated data or inter-individual differences, respectively. Assumptions of linearity, homoscedasticity, and normality of residuals were assessed for each predictor variable. Correlation coefficients for RMCORR (*r-rm)* and Pearson's correlation coefficients (*r*) were interpreted as: trivial (<0.1), small (0.1–0.3), moderate (0.3–0.5), large (0.5–0.7), very large (>0.7) (47). Significance was reported as *p* < 0.05. Workload variables were assessed corresponding to the week of the testing procedures as well as time lagged by one week to allow for causal insights on dependent variables. Workload – tendon structure and workload – neuromuscular performance associations were calculated and reported as between-subject correlations (*r*) and within-subject correlations (*r-rm*). Plots for each *r-rm* association are provided in Supplementary Figures 1–4.

## 3. Results

ICC values and 95% confidence intervals for patellar tendon thickness was 0.97 (0.92–0.99, *excellent*), echogenicity was 0.81 (0.45–0.95, *poor to excellent*), and stiffness was 0.99 (0.97–1.00, *excellent*). SEM values for patellar tendon thickness was 0.03 mm (0.15 mm–0.04 mm), echogenicity was 2.95 AU (1.51 AU–5.02 AU), and stiffness was 2.53 (0.00–4.38 N/m). Descriptive statistics for weekly workloads, neuromuscular performance, and patellar tendon structure are reported in Tables 1A–C, respectively.

**Table 1A:** Descriptive statistics - weekly workloads.

Week	Jump Count	sRPE	WLeff	Jump Count −1	sRPE −1	WLeff −1
1	363.19 ± 157.97	1683.62 ± 558.03	0.22 ± 0.07	N/A	N/A	N/A
2	365.31 ± 193.46	1846.25 ± 894.7	0.18 ± 0.1	363.19 ± 157.97	1683.62 ± 558.03	0.22 ± 0.07
3	316.28 ± 196.99	1380.4 ± 774.82	0.24 ± 0.07	365.31 ± 193.46	1846.25 ± 894.7	0.18 ± 0.1
4	N/A	N/A	N/A	316.28 ± 196.99	1380.4 ± 774.82	0.24 ± 0.07
5	233.5 ± 135.72	1153.75 ± 569.59	0.22 ± 0.08	N/A	N/A	N/A
6	359.81 ± 176.39	2034.88 ± 860.81	0.18 ± 0.08	233.5 ± 135.72	1153.75 ± 569.59	0.22 ± 0.08
7	219.44 ± 136.78	1073.32 ± 587.12	0.21 ± 0.09	359.81 ± 176.39	2034.88 ± 860.81	0.18 ± 0.08
8	305.88 ± 163.91	1475.88 ± 520.28	0.22 ± 0.1	219.44 ± 136.78	1073.32 ± 587.12	0.21 ± 0.09
9	454.62 ± 172.36	2618.75 ± 631.47	0.18 ± 0.06	305.88 ± 163.91	1475.88 ± 520.28	0.22 ± 0.1

sRPE, session rating of perceived exertion; wLeff, workload efficiency; NA, no data were available. Time lagged variables are indicated with a “-1”.

**Table 1B:** descriptive statistics - weekly performance testing.

Week	Countermovement Jump			Drop Jump		
	Jump Height (cm)	RSImod (AU)	Peak Force (N)	Jump Height (cm)	RSI (AU)	Peak Force (N)
1	54.74 ± 11.58	1.31 ± 0.28	11.33 ± 1	54.15 ± 10.64	2.52 ± 0.67	15.15 ± 3.87
2	55.25 ± 9.60	1.44 ± 0.3	11.21 ± 1	52.76 ± 7.49	2.72 ± 0.65	16.23 ± 3.77
3	56.77 ± 10.24	1.37 ± 0.27	11.33 ± 0.84	55.22 ± 9.96	2.58 ± 0.57	14.74 ± 1.53
4	NA	NA	NA	NA	NA	NA
5	55.47 ± 9.96	1.34 ± 0.26	11 ± 0.75	54.61 ± 8.97	2.53 ± 0.47	15.62 ± 3.12
6	55.93 ± 8.15	1.45 ± 0.22	11.4 ± 0.93	55.83 ± 8.46	2.67 ± 0.6	14.91 ± 2.75
7	54.56 ± 10.06	1.41 ± 0.34	11.49 ± 0.97	52.20 ± 7.82	2.64 ± 0.76	15.2 ± 1.9
8	57.12 ± 10.57	1.46 ± 0.25	11.31 ± 1.07	53.87 ± 8.84	2.67 ± 0.65	14.51 ± 1.89
9	56.52 ± 10.36	1.29 ± 0.39	11.18 ± 1.13	55.04 ± 6.65	2.73 ± 1.01	14.62 ± 3.15

RSImod, reactive strength index modified; RSI, reactive strength index; NA, no data were available.

**Table 1C:** Descriptive Statistics - Weekly Patellar Tendon Structure.

Week	Dominant Patellar Tendon			Non-Dominant Patellar Tendon		
	Thickness (mm)	Echogenicity (AU)	Stiffness (N/m)	Thickness (mm)	Echogenicity (AU)	Stiffness (N/m)
1	4.62 ± 0.86	69.56 ± 12.32	876.06 ± 99.36	4.04 ± 0.9	67.46 ± 9.19	805.44 ± 121.25
2	4.4 ± 0.92	70.87 ± 9.99	876.69 ± 104.76	4.36 ± 0.78	63.27 ± 9.86	794.44 ± 110.72
3	4.42 ± 0.71	70.76 ± 9.7	847.31 ± 120.49	4.37 ± 0.8	67.28 ± 10.16	815.94 ± 95.3
4	NA	NA	NA	NA	NA	NA
5	4.47 ± 0.65	61.85 ± 13.25	871.55 ± 94.79	4.45 ± 0.63	66.15 ± 10.45	851.64 ± 94.22
6	4.69 ± 0.68	65.22 ± 10.7	889.19 ± 101.18	4.13 ± 0.62	65.99 ± 9.48	846.25 ± 104.15
7	4.52 ± 0.96	66.46 ± 12.2	856.93 ± 135.98	4.09 ± 0.75	67.46 ± 10.82	863.2 ± 112.86
8	4.39 ± 0.68	56.06 ± 9.11	852.75 ± 114.21	4.05 ± 0.59	60.09 ± 7.47	837.69 ± 128.63
9	4.24 ± 0.64	58.1 ± 9.99	854.88 ± 99.14	4.41 ± 0.82	59.95 ± 8.95	809.31 ± 128.69

AU, arbitrary units; NA, no data were available.

### 3.1. Neuromuscular performance

**Figure 4:**
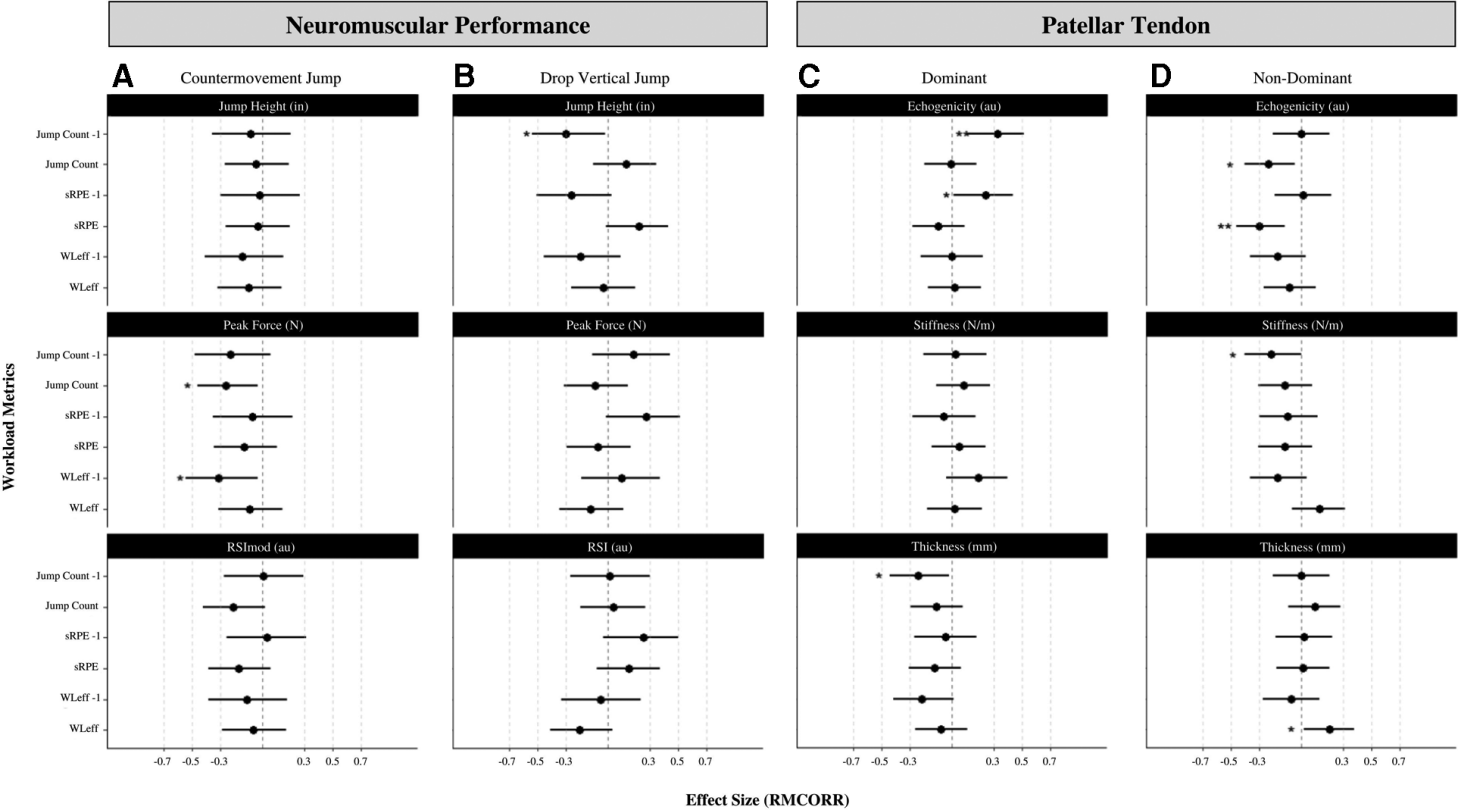
Effect sizes of RMCORR between (**A**) countermovement jump and workload, (**B**) drop vertical jump and workload, (**C**) dominant patellar tendon and workload, (**D**) non-dominant patellar tendon and workload. *p < 0.05, **p < 0.01. sRPE, session rating of perceived exertion; WLeff, workload efficiency, −1 indicates time-lagged variable.

**Figure 5:**
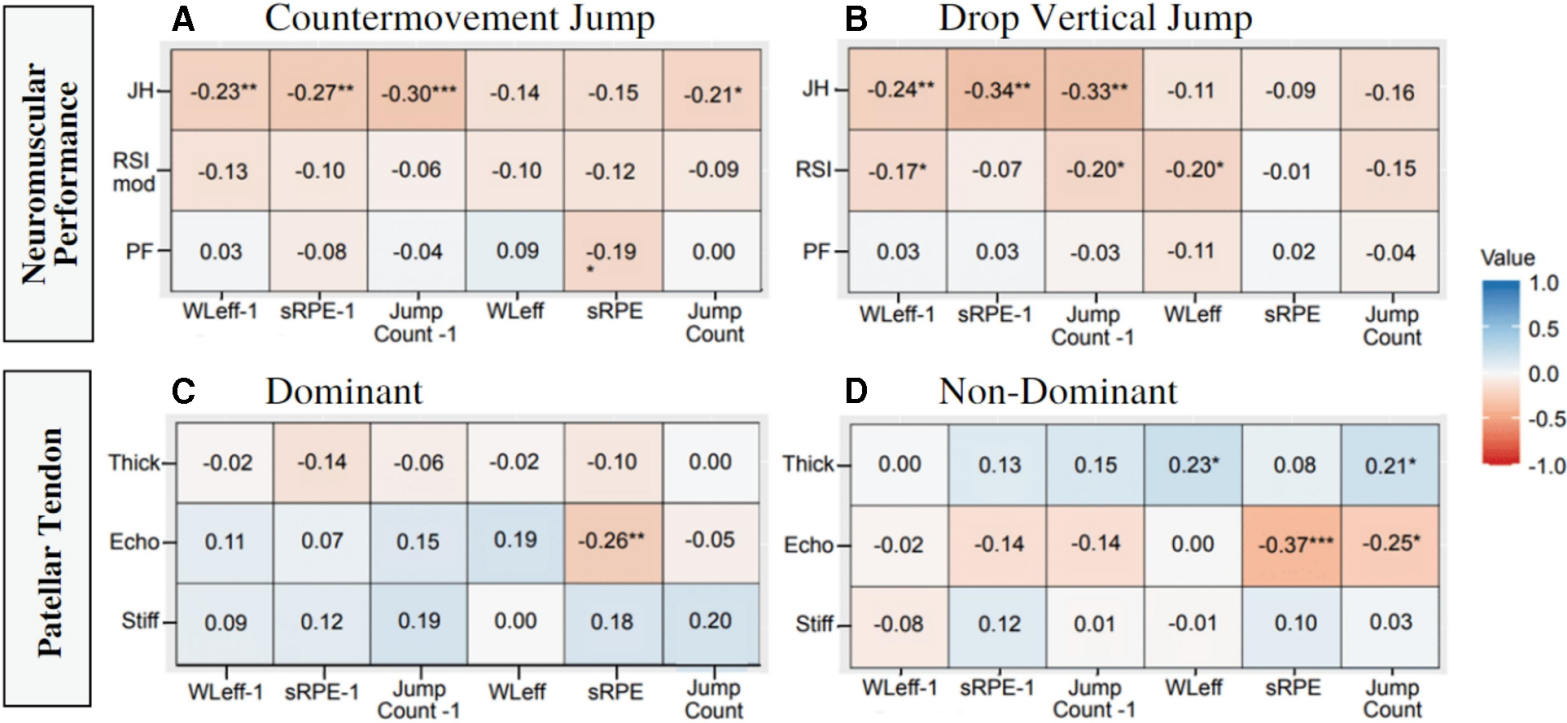
Pearson's correlation coefficient matrix between (**A**) countermovement jump and workload, (**B**) drop vertical jump and workload, (**C**) dominant patellar tendon and workload, (**D**) non-dominant patellar tendon and workload. *p < 0.05, **p < 0.01, ***p < 0.001. sRPE, session rating of perceived exertion; WLeff, workload efficiency, −1 indicates time-lagged variable.

Results for RMCORR are presented in Figures 4A,B for countermovement jump and drop vertical jump, respectively. Significant associations were exhibited between countermovement jump peak force and jump count (*r-rm *= −0.26; *small negative*, *p* = 0.02) of the current week as well as workload efficiency lagged (*r-rm *=* *−0.31; *moderate negative*, *p* = 0.03). No other significant associations were established for countermovement jump performance. Drop vertical jump height exhibited a significant association with jump count lagged (*r-rm *= −0.30, *moderate negative*. *p* = 0.03). No other drop vertical jump variables were associated with any workload metrics. Pearson's correlation coefficients are presented in Figure 5.

### 3.2. Patellar tendon structure

Results for patellar tendon structure are reported in Figures 4B,C representing dominant and non-dominant limb, respectively. Echogenicity of the dominant limb exhibited significant associations with jump count lagged (*r-rm *= 0.32; *moderate positive*. *p* = 0.003) and sRPE lagged (*r-rm *= 0.24; *moderate positive*. *p* = 0.03). Thickness of the dominant limb exhibited a significant association with jump count lagged (*r-rm *= −0.24; *small negative*. *p* = 0.03). No significant associations were exhibited in stiffness of the dominant limb and any workload metrics. Echogenicity of the non-dominant limb exhibited significant associations with jump count (*r-rm *= −0.23; *small negative*. *p* = 0.01) and sRPE (*r-rm *= −0.30; *moderate negative*. *p* = 0.001). Thickness of the non-dominant limb exhibited a significant association with workload efficiency (*r-rm *= 0.20; *small positive. p* = 0.03). Stiffness of the non-dominant limb exhibited a significant association with jump count lagged (*r-rm *= −0.21; *small negative*. *p* = 0.04). Pearson's correlation coefficients are presented in Figure 5.

## 4. Discussion

The overall purpose was to examine associations within the mechanical and psychophysiological load-response pathway between athlete loads, patellar tendon properties, and neuromuscular performance in collegiate men volleyball players. In partial support of the original hypotheses, significant *small to moderate* within-subject associations were reported through the mechanical load-response pathway, but not the psychophysiological load-response pathway. Further, *small to moderate* between-subject associations were reported in both load-response pathways with neuromuscular performance as the outcome. Also, *small to moderate* within- and between-subject associations were reported through both load-response pathways with patellar tendon properties as the outcome.

### 4.1. Neuromuscular performance

Accumulated exposure may result in fatigue, which is characterized by decrements in force production and kinematic alterations in movement strategy. Previous literature has demonstrated sensitivity of RSImod and flight time-to-contraction time ratio to indicate fatigue when exposed to high workloads (16, 48). However, current findings were not in support of those previously reported as a lack of intra-individual associations with RSI, RSImod, and workload metrics existed. Interestingly, a moderate negative intra-individual association between countermovement jump peak force and workload efficiency lagged was shown indicating a tradeoff of decreased force with a concomitant increase in workload efficiency of the previous week. This may have been influenced by training decisions outside of the monitored sport activity (e.g., resistance training). Considering workload efficiency is a ratio, changes in constituent parts relative to one another may influence the outcome. For example, if a higher increase in external workload relative to internal workload exists yet both parameters increase, workload efficiency will still improve. Perhaps this disparity in performance impairments is more affected by mechanical fatigue than perceived exertion. However, underlying contextual factors may exist and considering a lack of association between internal and external workload independently, whether this is an adaptation or not remains inconclusive.

Small to moderate negative associations were exhibited between countermovement jump height and drop vertical jump height with athlete workload metrics. In support of previous literature reporting variation in jumping demands is dependent upon player role, small negative associations between RSI and load metrics were exhibited (49). In the current analysis, athletes with lower jump counts displayed higher jump heights and higher reactive strength capacity. Back row players are characterized by higher jumping volumes during training and competition compared to front row players, who perform fewer jumps yet require higher intensity jumps for tasks such as blocking and spiking (50) A few possibilities exist to explain the small effect sizes seen in the current research. Slow (>250 ms) stretch-shortening cycle movements require unique force expression compared to fast (<250 ms) stretch-shortening cycle movements and may exhibit unique adaptations (51). A negative association with drop vertical jump height, but not countermovement jump height, may indicate fast stretch shortening cycle performance is impaired following higher workloads. Previous findings suggest countermovement jump height may remain stable as a result of technical alterations in order to maintain performance outcomes (52). The shorter duration movement may be more sensitive to fatigue and not provide adequate time for compensation. Another possible explanation is that a non-linear association exists between loads and neuromuscular performance indicators. Exposure and response varies across individuals and across time and thus, may be better supported as a non-linear association (53). More complex analysis techniques such as linear mixed models (54, 55) or time to event analysis (56, 57) may be suitable to address potential insights into individual slopes and time varying associations.

### 4.2. Patellar tendon structure

Habitual plyometric loading may elicit positive tendon adaptations provided the stimulus is adequate and ample recovery afforded (29, 58). Conversely, maladaptation may occur in response to suboptimal loading patterns leading to hypoechoic regions and increases in thickness (26). Tendon thickening has been established as an adaptive mechanism allowing for more efficient transfer of mechanical energy (59) with supporting evidence that patellar tendon thickness is associated with higher countermovement jump height (34). Acute inflammatory responses may partially explain the early stages of tendon pathology (60). Preseason training has been characterized by the highest training loads compared to other phases of competition in collegiate volleyball athletes (61). The current data support a decrease in thickness, which may indicate a reduction in inflammation from the high stress of preseason training. Thickness of the non-dominant patellar tendon exhibited a small positive association with workload efficiency indicating a decreased perception of effort with a reduction in thickness possibly influenced by a reduction in inflammation. Psychophysiological assessments, such as sRPE, are influenced by the current status of the athlete. Further, inflammation is linked to pain in tendinopathy literature possibly influencing perceived exertion (60, 62). In the dominant patellar tendon, an increase in echo intensity and decrease in thickness were associated with increased jump count lagged. Increase in echogenicity was associated with increased sRPE lagged while echogenicity of the non-dominant patellar tendon exhibited small and moderate negative associations with jump count and sRPE. Ground reaction forces upwards of 4× bodyweight can be experienced in a landing following an attack in volleyball (63) Attackers have been shown to prefer unilateral landing characterized by landing contact of the opposite side of the swing arm 68% of the time (64). The aforementioned findings may explain the observed inter-limb discrepancies in the current study and emphasize the need to develop more precise methods of measuring mechanical load (65).

Negative inter-individual associations were exhibited between echogenicity of non-dominant and dominant patellar tendon and sRPE, suggesting perceived exertion may be impacted by patellar tendon structure. A negative inter-individual association was exhibited between echogenicity of the non-dominant patellar tendon and jump count, suggesting a healthier tendon on the non-dominant limb in those who perform fewer average jumps. Overuse injury is more dependent upon magnitude of loading than frequency of loading (14). Thus, frequent unilateral landing of the non-dominant side in conjunction with large ground reaction forces when landing from higher jump heights may explain the observed differences in dominant compared to non-dominant adaptations.

Stiffness of the non-dominant patellar tendon exhibited a small negative intra-individual association with jump count lagged. Pathological patellar tendon tends to exhibit a decreased stiffness, which may lead to higher strain in the tendon (26). High strain is characterized by larger deformation when exposed to mechanical forces, which may be responsible for risk of structural damage (2). Intra-individual associations with time lagged external load and stiffness and with current weak external load and echogenicity may indicate temporal differences in the onset of structural changes in response to load. To date, patellar tendon pathology has been identified through unique characteristics present in pathological conditions (22, 26, 66). Limited research has examined the latency of structural and mechanical abnormalities in patellar tendinopathy (30). While abnormalities coexist in pathological conditions, the time course of onset and recovery may differ. The current research provides novel insight into intra-individual associations with external and internal loads, neuromuscular performance, and patellar tendon structural properties. Further, the findings support the utility of assessing and monitoring ultrasound imaging metrics relevant to patellar tendinopathy in volleyball athletes.

### 4.3. Future directions and limitations

Monitoring athlete load to mitigate risk of overuse is worthwhile in order to determine optimal load prescription. However, the utility of ultrasound image analysis to assess tendon pathology remains in question. The current analysis provides support for associations between athlete load and patellar tendon structure, but requires further evidence to identify relevant contextual factors. It is recommended that the robustness of the current structural properties along with other relevant image metrics of tendon pathology be examined. Since tendon pathology demonstrates an array of abnormalities with each likely occurring on a unique timeline, investigation into the time course and development of abnormalities may help guide detection and risk mitigation.

The current research is not without limitations. Time lagged workload metrics included 7 of the 9 time points with additional missing data depending upon the individual. Although RMCORR is robust to missing data, a complete dataset would provide stronger evidence. Further, RMCORR is limited to linear associations and although suitable for addressing the fundamental research question presented in the current research, more complex modeling could be used to evaluate time-varying, non-linear trends in load exposure, training responses, and associations. Quantifying mechanical load experienced in the tissue is theoretically crucial to understanding adaptations of the corresponding tissue. This poses limitations on interpretation and may reduce the effectiveness of determining associations with structural adaptations. The accelerometers used in the current study are worn close to the athlete's center of mass and detect movements as a system. Jumping and landing tasks often impose unilateral demands that cannot be accurately depicted; therefore, reliance on systemic, whole-body surrogate measures may account for the small effects observed. Injury data was unavailable at the time of this data collection. Previous injury is known as an influential factor for future pathology, particularly in patellar tendinopathy, and is recommended to include as an intrinsic risk factor when evaluating injury risk and mitigation strategies. Asymptomatic tendon as well as unreported symptomatic tendon may influence these findings and is recommended to be further investigated.

## 5. Conclusion

Patellar tendon pathology in symptomatic and asymptomatic athletes may contribute to altered physical and psychophysiological impairments. Thus, mechanisms of patellar tendon pathology remain a topic of discussion. Further, the utility of ultrasound imaging to assess and monitor patellar tendon structure and associations with injury risk remains inconclusive. The current study provides evidence of associated changes in neuromuscular performance and patellar tendon structure in response to athlete load, and provides insight into future applications of performance testing and imaging techniques. Material and morphological adaptations may be useful in assessing change in patellar tendon to identify onset of overuse injury. It is recommended that future research investigate non-linear associations and individual analysis of these associations.

## Data Availability

The raw data supporting the conclusions of this article will be made available by the authors, without undue reservation.

## Supplementary material

The Supplementary Material for this article can be found online at: https://www.frontiersin.org/articles/10.3389/fspor.2023.1065470/full#supplementary-material. Supplementary Figure 1. RMCORR plots for workloads and countermovement jump (A) jump height (JH), (B) RSImod, and (C) peak force (PF). sRPE = session rating of perceived exertion, WLeff = workload efficiency, -1 indicates time-lagged variable. Supplementary Figure 2. RMCORR plots for workloads and drop vertical jump (A) jump height (JH), (B) RSI, and (C) peak force (PF). sRPE = session rating of perceived exertion, WLeff = workload efficiency, -1 indicates time-lagged variable. Supplementary Figure 3. RMCORR plots for workloads and dominant patellar tendon (A) thickness (Thick) (B) echogenicity (Echo), and (C) stiffness (Stiff). sRPE = session rating of perceived exertion, WLeff = workload efficiency, -1 indicates time-lagged variable. Supplementary Figure 4. RMCORR plots for workloads and non-dominant patellar tendon (A) thickness (Thick) (B) echogenicity (Echo), and (C) stiffness (Stiff). sRPE = session rating of perceived exertion, WLeff = workload efficiency, -1 indicates time-lagged variable.
